# Enterotoxigenic *Bacteroides fragilis* induces the stemness in colorectal cancer via upregulating histone demethylase JMJD2B

**DOI:** 10.1080/19490976.2020.1788900

**Published:** 2020-07-20

**Authors:** Qian-Qian Liu, Chun-Min Li, Lin-Na Fu, Hao-Lian Wang, Juan Tan, Yun-Qian Wang, Dan-Feng Sun, Qin-Yan Gao, Ying-Xuan Chen, Jing-Yuan Fang

**Affiliations:** Division of Gastroenterology and Hepatology, Key Laboratory of Gastroenterology and Hepatology, Ministry of Health, State Key Laboratory for Oncogenes and Related Genes, Shanghai Institute of Digestive Disease, Renji Hospital, School of Medicine, Shanghai Jiao Tong University, Shanghai, China

**Keywords:** Enterotoxigenic *Bacteroides fragilis*, colorectal cancer, stemness, histone demethylase, JMJD2B

## Abstract

The enrichment of Enterotoxigenic *Bacteroides fragilis* (ETBF) has been identified in CRC patients and associated with worse prognosis. Cancer stem cells (CSCs) play essential roles in CRC development. However, whether ETBF is involved in CSCs regulation is unknown. To clarify the role of ETBF in CSCs properties, we performed extreme limited dilution assays (ELDA) in nude mice injected with ETBF-treated or untreated CRC cells subcutaneously, tumor organoids culture in azoxymethane (AOM) mouse model after gavaging with or without ETBF, and cell sphere formation assay after incubating CRC cell lines with or without ETBF. The results indicated that ETBF increased the stemness of CRC cells *in vivo* and *in vitro*. Furthermore, ETBF enhanced the expression of core stemness transcription factors Nanog homeobox (NANOG) and sex determining region Y-box 2 (SOX2). Histone H3 Lysine 9 trimethylation (H3K9me3) is critical in regulating CSCs properties. As an epigenetic and transcriptional regulator, JmjC-domain containing histone demethylase 2B (JMJD2B) is essential for embryonic stem cell (ESC) transformation and H3K9me3 demethylation. Mechanistically, ETBF infection significantly upregulated JMJD2B levels in CRC cell lines and nude mice xenograft model. JMJD2B epigenetically upregulated NANOG expression via demethylating its promoter H3K9me3, to mediate ETBF-induced stemness of CRC cells. Subsequently, we found that the Toll-like receptor 4 (TLR4) pathway, activated by ETBF, contributed to the enhanced expression of JMJD2B via nuclear transcription factor nuclear factor of activated T cells 5 (NFAT5). Finally, in human CRC samples, the amount of ETBF positively correlated with nuclear NFAT5, JMJD2B, and NANOG expression levels. In summary, ETBF upregulated JMJD2B levels in a TLR4-NFAT5-dependent pathway, and played an important role in stemness regulation, which promoted colorectal carcinogenesis.

## Introduction

Colorectal cancer (CRC) is the third most common cancer worldwide.^1,2^ The gut microbiota, along with genetic and other environmental factors, contributes to the carcinogenesis of CRC. Evidence for microbiota involvement in colorectal carcinogenesis can be found in animal models of intestinal carcinogenesis, where both antibiotic-treated conventional and germ-free mice models developed cancer after gavage with fecal samples from patients with CRC. Enterotoxigenic *Bacteroides fragilis* (ETBF) is a human colonic symbiotic anaerobe that is prevalent in up to 50% of the healthy individuals.^3^ A link between ETBF and inflammatory bowel disease,^4^ as well as CRC, has been established. Recent clinical data have shown that ETBF is significantly enriched in stool and mucosa samples from patients with CRC compared with those in the healthy controls.^5,6^ Moreover, the prevalence of ETBF in CRC tissues is associated with poor prognosis.^3^ Researchers have shown that mice implanted with ETBF and *Escherichia coli* are more likely to develop CRC and die.^7^ Furthermore, ETBF might promote colorectal carcinogenesis by activating the nuclear factor kappa B (NF-κB) or Wnt signaling pathways,^8,9^ increasing polyamine metabolism,^10^ inducing DNA damage,^11^ and activating Th17 adaptive immunity.^12,13^ However, ETBF’s possible mechanisms in CRC remain unclear.

Cancer stem cells (CSCs), also called tumor-initiating cells (TICs), are a subset of tumor cells that exhibit self-renewal ability.^14,15^ Colorectal CSCs are positively associated with higher recurrence rates.^16,17^ CRC with stem cell signatures, such as CD44 positivity, NANOG positivity, or SOX2 positivity, have been associated with resistance to several anticancer drugs, for example, cisplatin, 5-fluorouracil (5-FU), irinotecan, and oxaliplatin.^18–20^ Therefore, advancing our understanding of the molecular properties and signaling pathways unique to CSCs is vital to develop a new generation of targeted and effective therapies for CRC. The self-renewal of CSCs results from a complex interplay between gene regulation and the environment. A recent study published in *Science* reported that acidophilic infection can enhance the activity of intestinal stem cells.^21^ *Enterococcus faecalis* colonized in the intestinal epithelium of mice promoted the development of CRC by increasing the expression of tumor stem cell markers.^22^ Study has also shown that nonpathogenic *E. coli* upregulates the expression of CSC markers, thus engendering tumorigenic stemness in host cells.^23^ Thus, bacterial infections might increase the incidence of CRC by stimulating stem cell activity. However, the regulation by ETBF of CSC properties remains largely unknown.

In the present study, we investigated whether ETBF induces stemness during tumorigenesis of CRC and the molecular mechanisms involved. We found that JmjC-domain containing histone demethylase 2B (JMJD2B), which is induced by ETBF in a Toll-like receptor (TLR) 4-nuclear factor of activated T-cells 5 (NFAT5)-dependent manner, plays an important role in the stemness of human CRC cells, which could promote the expression of NANOG by binding and removing the inhibitory H3K9me3 marks on the *NANOG* promoter region. This study illustrates a new mechanism of promoting the development of CRC mediated by a specific pathogen.

## Results

### *ETBF promotes colorectal tumorigenesis* in vivo *and is enriched in CRC patients with advanced TNM stage*

It was reported that ETBF-induced inflammatory colitis progressed to colon tumorigenesis in Min mice.^12^ To demonstrate the effects of ETBF on CRC tumorigenicity *in vivo*, we established two different animal models, the AOM-induced sporadic CRC model and the nude mouse xenograft model. In the AOM model, after microbiota depletion using antibiotics (Supplementary Figure S1A), NTBF or ETBF (Supplementary Figure S1B) were introduced to the mice, accompanied by intraperitoneal injection of AOM. We observed that treatment with ETBF significantly increased the number and volume of intestinal tumors in the AOM-injected mice, as compared with the control group and NTBF-gavaged group (Supplementary Figure S1 C, D). A similar role of ETBF was found in the xenograft tumors in nude mice (Supplementary Figure S1E). To identify the clinical significance of ETBF, we performed real-time PCR to quantify the abundance of ETBF in CRC tissue from 56 patients with CRC with different clinicopathological features. According to their ETBF abundance, the patients with CRC were divided into two groups (26 ETBF-low or 30 ETBF-high expression). The correlations between ETBF abundance and clinicopathological factors (age, sex, tumor size, location, TNM stage, and histology) were evaluated and are presented in Table 1. TNM stage III and IV in the ETBF-high group were more frequent than in the ETBF-low group (*P* = .025). Relative ETBF abundance in TNM stage III and IV is higher than that in TNM stage I and II (Supplementary Figure S1 F). Collectively, these results confirmed the pro-tumorigenicity effect of ETBF in CRC, and suggested that ETBF abundance might be positively related with poor prognosis.

**Table 1.** Characteristics of CRC patients according to ETBF status.

		The expression of ETBF		
Clinical characteristics	Patients (N = 56)	Low (N = 26)	High (N = 30)	*P* value*
Gender- no. (%)				.789
Man	30 (53.6)	13 (50)	17 (57)	
Women	26 (46.4)	13 (50)	13 (43)	
Age (mean, y)	67.5	67.61	67.3	.964
Tumor size (mean, cm)	3.78	3.81	3.75	.899
Tumor location				.293
Left colon	26 (46.4)	14 (53.8)	12 (40)	
Right colon	28 (50)	12 (46.2)	16 (53.3)	
Rectum	2 (3.6)	0 (0)	2 (6.7)	
TNM- no. (%)				.025
I–II	32 (57.1)	19 (69.2)	13 (46.7)	
III–IV	24 (42.9)	7 (30.8)	17 (53.3)	
Pathological type				.066
Tubular adenocarcinoma	47 (83.9)	19 (73.1)	28 (93.3)	
Mucinous adenocarcinoma	9 (16.1)	7 (26.9)	2 (6.7)	
Histo-differentiation				.094
Well/moderate	53 (94.6)	23 (88.5)	30 (100)	
Poor differentiation	3 (5.4)	3 (11.5)	0 (0)	

*: Statistical significance was determined by χ^2^-test

### *ETBF induces stem cell-like properties* in vitro *and* in vivo

**Figure 1.**
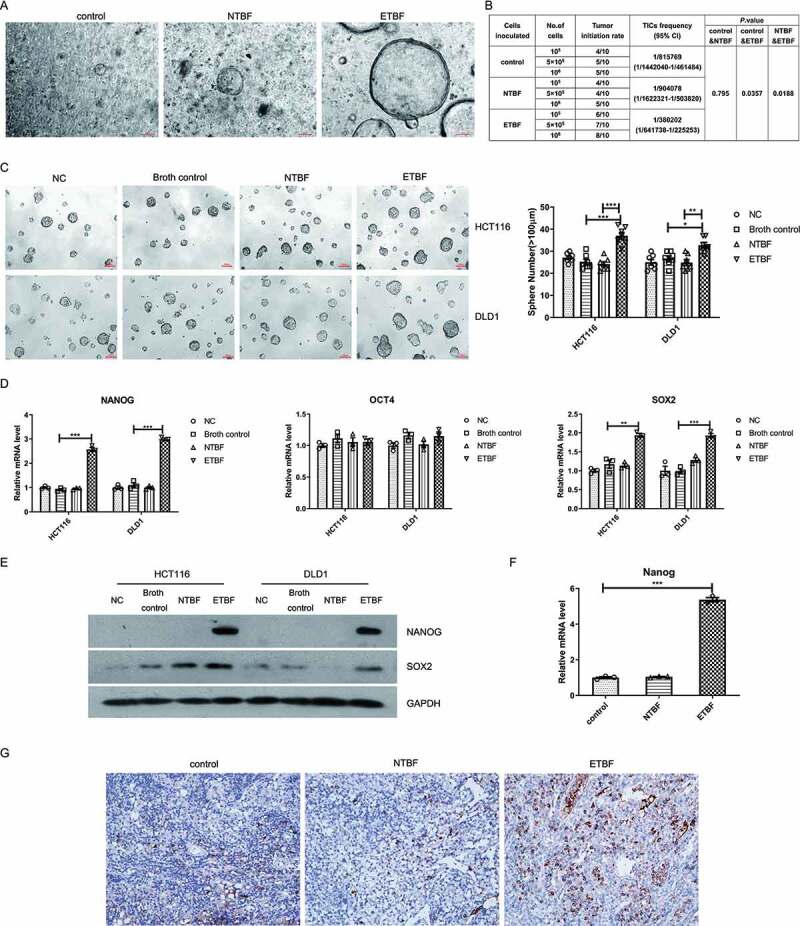
ETBF induces stem cell-like properties *in vitro* and *in vivo*. A, Representative images of organoids obtained from tumors of NTBF/ETBF-gavaged mice of the AOM model. Circles in graph indicate individual organoid cultures. Scale bar, 100 µm. B, The extreme limiting dilution analysis (ELDA) to calculate the tumor initiating CSC frequency after transplantation of limiting numbers of HCT116 cells co-cultured with NTBF or ETBF into nude mice. C, HCT116 and DLD1 cells (1000 cells/200 μL per well) were co-cultured with cell culture medium (NC) or bacteria culture medium (Broth control) as controls, and ETBF or NTBF for 6 h, then changed to serum-free medium for an additional 5 days. Representative tumorsphere images were acquired and the number of tumorspheres (diameter ≥ 100 μm) was quantified. Scale bar, 100 μm. D, The expression of stemness markers or maintainers were detected by real-time PCR in cancer cells co-cultured with ETBF. E, Western blotting results for NANOG and SOX2 was performed. F, The expression of *Nanog* was detected using real-time PCR in organoids obtained from tumors of NTBF/ETBF-gavaged mice in the AOM model. G, Representative immunohistochemistry of NANOG protein in xenograft tumor tissues. 400 × magnifications. Data are expressed as the mean ± SEM from three independent experiments. Statistical significance was determined by ANOVA. *, *P < *.05; **, *P < *.01; ***, *P < *.001.

Recent studies showed that ETBF promoted CRC through affecting proliferation,^9^ apoptosis,^24^ metabolism,^10^ and immunity.^25,26^ However, the role of ETBF in CSCs properties is unknown. In order to investigate the effect of ETBF in stem cell-like phenotype regulation in CRC *in vivo*, we performed intestinal organoid culture using individual CRC specimens from AOM-injected mice and extreme limited dilution assays (ELDA^27^) using xenograft tumors in nude mice, respectively. We found that the growth and size of the tumor organoids increased in the ETBF-gavaged group compared with those of the control, or NTBF-gavaged groups in AOM-injected mice (Figure 1A). In the xenograft model, transplantation of limiting numbers of HCT116 cells into nude mice induced a 2.14-fold increase in the absolute number of TICs in the tumor from ETBF-stimulated mice (1/380202) compared with those from the controls (1/815769) (Figure 1B).

To further evaluate the role of ETBF in regulating stemness *in vitro*, we co-cultured CRC cell lines HCT116 and DLD1 with ETBF, and then analyzed their tumorsphere formation capacity. ETBF significantly increased the number of spheres (diameter ≥ 100 μm) compared with the Broth control or NTBF group (Figure 1C). NANOG, SOX2, and Octamer-binding transcription factor 4 (OCT4) make up the core transcriptional network responsible for the regulation of stem cell self-renewal and pluripotency.^28,29^ Among them, Nanog may be the signaling hub that controls the other core ESC transcription factors.^30,31^ In this study, core transcription factors for CSCs, NANOG, SOX2 and OCT4 were compared by real-time PCR and western blotting. We found that NANOG and SOX2 were significantly elevated in the ETBF-infected cells, at both mRNA and protein level, while OCT4 showed no significant change (Figure 1D,E). In line with the above results, real-time PCR and histological staining also revealed that the tumor organoids collected from ETBF-gavaged C57BL/6 mice and tumors in the ETBF-stimulated nude mice expressed higher levels of NANOG than did in the controls (Figure 1F,G).

Thus, all these data indicated that ETBF increases the stemness of CRC cells *in vitro* and *in vivo*.

### JMJD2B epigenetically upregulates NANOG expression to mediate ETBF- induced stemness of CRC cells

**Figure 2.**
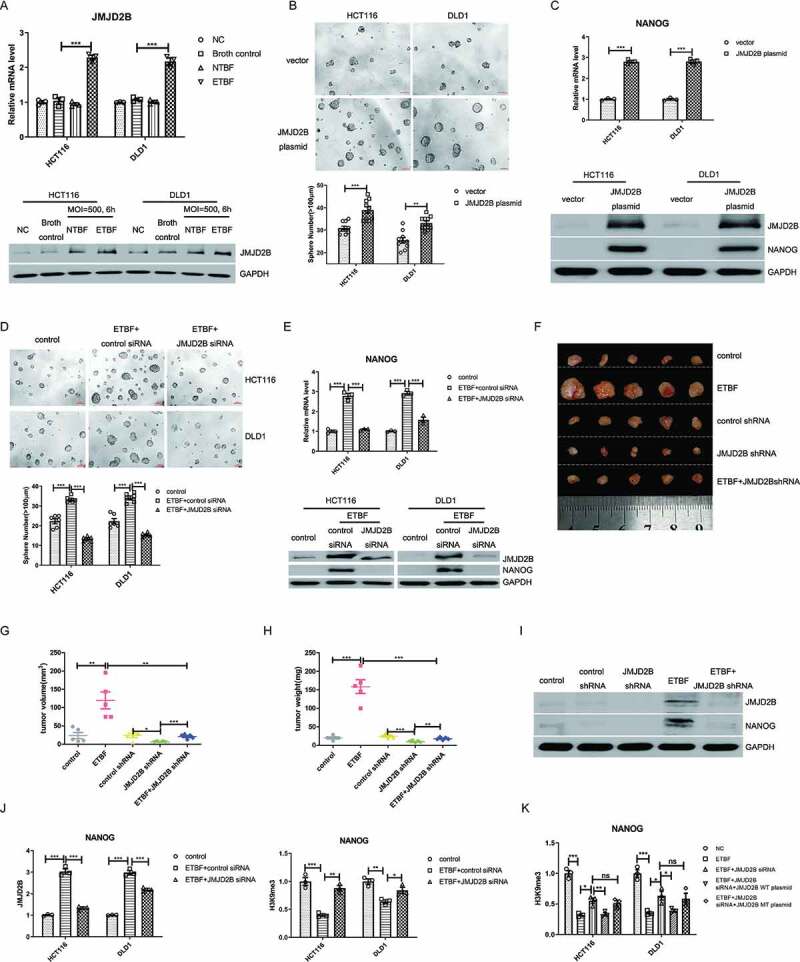
JMJD2B epigenetically upregulates NANOG expression to mediate ETBF-induced stemness of CRC cells. A, The expression of JMJD2B was measured using real-time PCR and western blotting in CRC cells co-cultured with ETBF. B, Representative tumorsphere images were acquired and the number of tumorspheres (diameter ≥ 100 μm) was quantified in CRC cells (1000 cells/200 μL per well) transfected with the JMJD2B overexpression plasmid. Scale bar, 100 μm. C, The effect of JMJD2B plasmid transfection on Nanog expression in HCT116 and DLD1 cells. D, ETBF-co-cultured HCT116 and DLD1 cells (1000 cells/200 μL per well) with either scrambled control or JMJD2B siRNA were subjected to tumorsphere formation assay, and the average number of tumorspheres (≥ 100  µm in diameter) was analyzed. E, Knockdown of JMJD2B could abolish the effect of ETBF on the upregulation of NANOG in HCT116 and DLD1 cells. F, Representative data of tumors in nude mice bearing HCT116 cells (1 × 10^6^ cells) in different groups. N = 5/group. G and H, Statistical analysis of tumor volume (G) and tumor weight (H) in different groups, n = 5/group. I, Western blotting of JMJD2B and NANOG from the representative xenograft tumor tissues in different groups. J, Effect of JMJD2B on the occupancies of H3K9me3 in the promoters of *NANOG* in HCT116 and DLD-1 cells by a ChIP assays (real-time PCR). K, Analysis of the levels of H3K9 tri-methylation binding to the *NANOG* promoter in HCT116 and DLD-1 cells to test whether the decrease in H3K9me3 intensity was depended on the lysine de-methylation activity of JMJD2B directly (real-time PCR). Data are expressed as the mean ± SEM from three independent experiments. Statistical significance was determined by ANOVA (A, D, E, G, H, J and K) and unpaired Student’s t test (B and C). *, *P < *.05; **, *P < *.01; ***, *P < *.001.

The gut microbiota affects host tissue H3 methylation patterns, suggesting a role for the gut microbiota as a driver of chromatin regulation.^32^ JMJD2B was identified as an H3K9me3/2 demethylase that activates target gene transcription. To analyze the role of JMJD2B in ETBF-induced stemness in CRC, we first observed JMJD2B expression after ETBF incubation. The results revealed that JMJD2B was significantly upregulated in the ETBF-infected cells (Figure 2A), whereas the protein levels of JMJD2A, JMJD2C, and JMJD2D, the other members of the JMJD2 family, were not induced (Supplementary Figure S2A). Next, we studied whether other CRC-related gut microbiota induced JMJD2B expression. Western blotting showed that the effect of ETBF in upregulating JMJD2B was the most obvious in CRC cells when compared with *E. coli, F. nucleatum*, and *C. symbiosum* (Supplementary Figure S2B). Given JMJD2B is a key rate-limiting enzyme that regulates stem cell activity,^33^ we further examined whether JMJD2B modulated CRC stemness. Tumorsphere cells possess the characteristics of CSCs when compared with the adherent cells; therefore, HCT116 and DLD1 cells were plated in a serum-free suspension culture system to allow tumorsphere formation. As expected, the levels of JMJD2B transcripts and proteins (Supplementary Figure S2 C) were enhanced in the tumorsphere cells compared with those in the adherent cells. Ectopic expression of JMJD2B (Supplementary Fig. S2D) signiﬁcantly promoted sphere formation (Figure 2B) and increased the expression of NANOG (Figure 2C) in HCT116 and DLD1 cells.

To address whether JMJD2B participates in ETBF-induced stemness, we analyzed the stemness properties in JMJD2B siRNA-transfected CRC cells co-cultured with ETBF. Silencing JMJD2B in the ETBF-infected cells inhibited sphere formation and NANOG expression (Figure 2D,E). The data indicated that ETBF might induce CRC stemness by increasing JMJD2B expression. In addition, the ETBF-induced pro-tumorigenicity effect was reversed by JMJD2B shRNA treatment in tumor-bearing mouse models, as shown by the reduced tumor weight and volume (Figure 2f-H). Moreover, knockdown of JMJD2B blocked ETBF-induced NANOG upregulation in the xenograft tumor tissues (Figure 2I). Thus, these data support the view that ETBF-induced colorectal cancer stemness is dependent on JMJD2B.

As shown in Figure 2E, a higher level of *NANOG* mRNA was detected in the ETBF-cocultured cells; while downregulation of JMJD2B suppressed the increase in *NANOG* transcription induced by ETBF. Furthermore, a decrease in the NANOG protein level was detected in JMJD2B-depleted HCT116 and DLD-1 cells co-cultured with ETBF (Figure 2E). In line with this, knockdown of JMJD2B led to a decrease in NANOG expression in the xenograft tumor tissues (Figure 2I). These data indicated that JMJD2B is involved in NANOG regulation in response to ETBF intervention. JMJD2B is a specific demethylase for histone H3K9me3/me2; therefore, we further analyzed the mechanism by which JMJD2B regulates NANOG. ChIP assays (Figure 2J, Supplementary Figure S2E) revealed a significant increase in JMJD2B binding to the *NANOG* promoter during ETBF infection, while this binding was significantly impaired after JMJD2B knockdown. Meanwhile, the H3K9me3 levels on the *NANOG* promoter significantly decreased under ETBF infection conditions and were restored after JMJD2B knockdown. To test whether the decrease in H3K9me3 intensity was catalyzed by JMJD2B directly, the siRNA-resistant JMJD2B wild-type plasmid (JMJD2B-WT) and the H189A/E191Q mutant plasmid (JMJD2B-MT), a catalytically inactive mutant without lysine de-methylation activity,^34,35^ were transfected into JMJD2B-knockdown cells under ETBF stimulation. In contrast to JMJD2B WT, a nonsignificant change in recruitment was detected in the JMJD2B MT group in JMJD2B-knockdown cells under ETBF stimulation (Figure 2K, Supplementary Figure S2F). These data indicated that JMJD2B-mediated regulation of Nanog is dependent on its demethylase activity.

These results indicated that JMJD2B transactivated *NANOG* by binding to its promoter and removing the transcriptionally repressive H3K9me3, thus mediated ETBF-induced stemness of CRC cells.

### NFAT5 is involved in ETBF-mediated stemness through upregulation of JMJD2B

**Figure 3.**
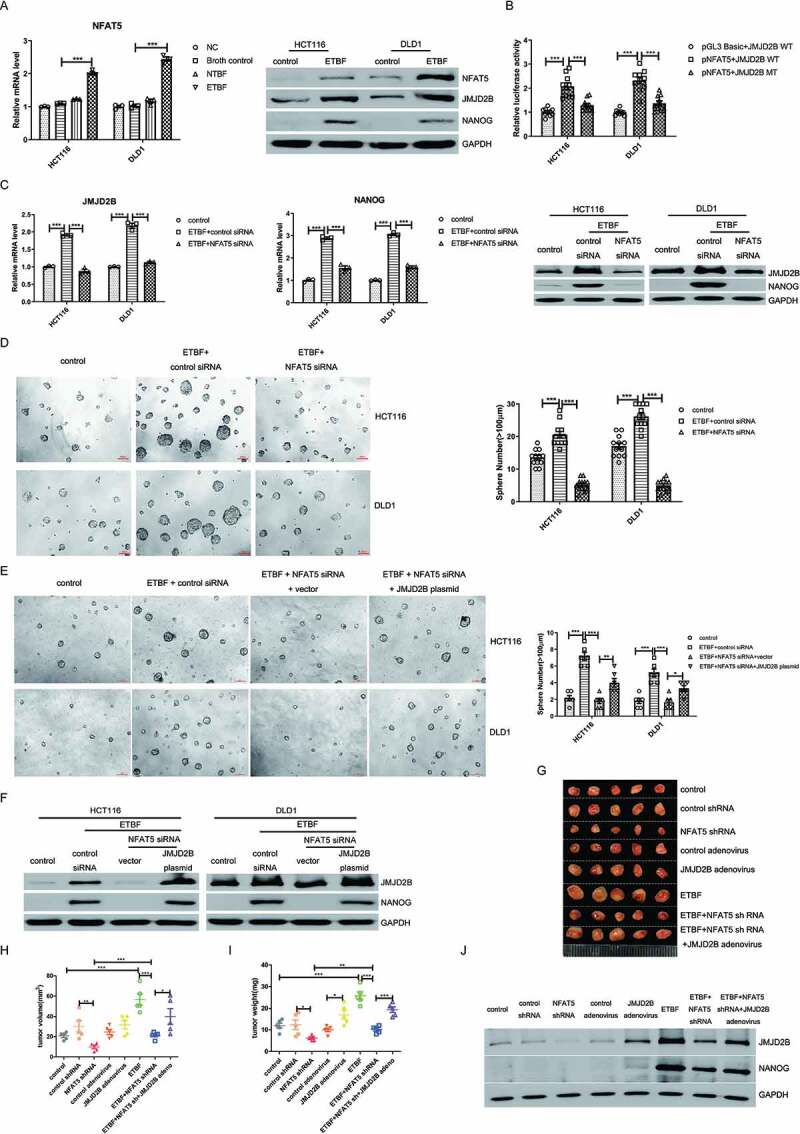
ETBF promotes the expression of JMJD2B through NFAT5. A, The expression of NFAT5 was measured by real-time PCR and western blotting in CRC cells co-cultured with ETBF (control: Broth control). B, Luciferase activity was measured in CRC cells transfected with the *NFAT5* overexpression plasmid or control plasmid. The luciferase JMJD2B recombinant plasmid containing wild-type or mutant binding sites for human *NFAT5* were used. The luciferase activity was normalized based on the control vector transfection. C, Real-time PCR and western blotting were performed to detect JMJD2B expression in HCT116 and DLD1 cells. The CRC cells were co-cultured with ETBF and transfected with NFAT5 siRNA. D, ETBF co-cultured HCT116 and DLD1 cells (1000 cells/200 μL per well) with either scrambled control or NFAT5 siRNA were subjected to tumorsphere formation assay, and the average number of tumorspheres (≥ 100 µm in diameter) were analyzed. E, The JMJD2B overexpression plasmid transfection could abolish the effect of NFAT5 siRNA on the reduction of tumorsphere formation in HCT116 and DLD1 cells (500 cells/200 μL per well). F, Western blotting was performed to detect the expression of NANOG in CRC cells. HCT116 and DLD1 cells were treated with NFAT5 siRNA, co-cultured with ETBF, and transfected with JMJD2B overexpression plasmid. G – I, Representative data of tumors (G) in nude mice bearing HCT116 cells (1 × 10^6^ cells) in the different indicated groups. Statistical analysis of tumor volume (H) and tumor weight (I) in different groups was performed, n = 5/group. J, Western blotting show the level of NFAT5, JMJD2B, and NANOG from the representative xenograft tumor tissues in the different indicated groups. Data are expressed as the mean ± SEM from three independent experiments. Statistical significance was determined by ANOVA. *, *P < *.05; **, *P < *.01; ***, *P < *.001.

To explore the mechanism by which ETBF induces upregulation of JMJD2B at both the mRNA and protein levels, bioinformatic software was used to predict transcription factor binding sites at promoter regions of *JMJD2B*. We found that the *JMJD2B* promoter region contained multiple binding sites for NFAT5 (Supplementary Fig. S3A). NFAT5 is a transcription factor that functions as a cell signaling molecule involved in complex adaptive systems. Interaction of lipopolysaccharide (LPS) with host cells through TLRs upregulates the expression of NFAT5.^36^ Therefore, we hypothesized that dysregulated NFAT5 expression might contribute to ETBF-increased JMJD2B expression. Real-time PCR and western blotting revealed that exposure of HCT116 and DLD1 cells to ETBF, but not to NTBF, could dramatically increase the expression of NFAT5 (Figure 3A), whereas the mRNA levels of its isoforms, *NFAT c1-c4*, were not significantly affected in both HCT116 and DLD1 cells (Supplementary Fig. S3B). Further, we conducted luciferase reporter assays and found that forced overexpression of *NFAT5* notably increased the luciferase activity of HCT116 and DLD1 cells transfected with a wild-type JMJD2B recombinant plasmid (JMJD2B-WT), but not with the mutant plasmid (JMJD2B-MT), which contains no binding sites for NFAT5 predicted by bioinformatic software (Figure 3B). These data indicated that NFAT5 could transcriptionally upregulate the expression of JMJD2B. Moreover, real-time PCR and western blotting revealed NFAT5 siRNA dramatically abrogated ETBF-induced upregulation of JMJD2B and NANOG (Figure 3C and Supplementary Fig. S3 C). In addition, sphere formation was reduced after knocking down NFAT5 in HCT116 and DLD1 cells co-cultured with ETBF, compared with that in the controls (Figure 3D). This effect was abolished in JMJD2B overexpression plasmid-transfected cells (Figure 3E, F).

In the CRC xenograft mouse models, HCT116 cells were inoculated into nude mice, followed by treatment with NFAT5 shRNA, ETBF co-culture, JMJD2B adenovirus, and other treatments. Tumor growth *in vivo* was determined by measuring the tumor volume and weight. As shown in Figure 3G-I, ETBF-induced tumor growth was significantly decreased by knocking down NFAT5, and these effects were reversed by JMJD2B adenovirus transduction in CRC-bearing nude mice. Furthermore, western blotting confirmed that knockdown of NFAT5 suppressed ETBF stimulated-JMJD2B and NANOG expression in the xenograft tumor tissues, and the NANOG decrease was efficiently rescued by JMJD2B overexpression (Figure 3J). Taken together, these results indicated that NFAT5 is involved in ETBF-mediated stemness via upregulation of JMJD2B, which participates in ETBF-mediated CRC tumorigenesis.

### ETBF induces CRC stemness through selectively activating the Toll-Like 4 pathway

**Figure 4.**
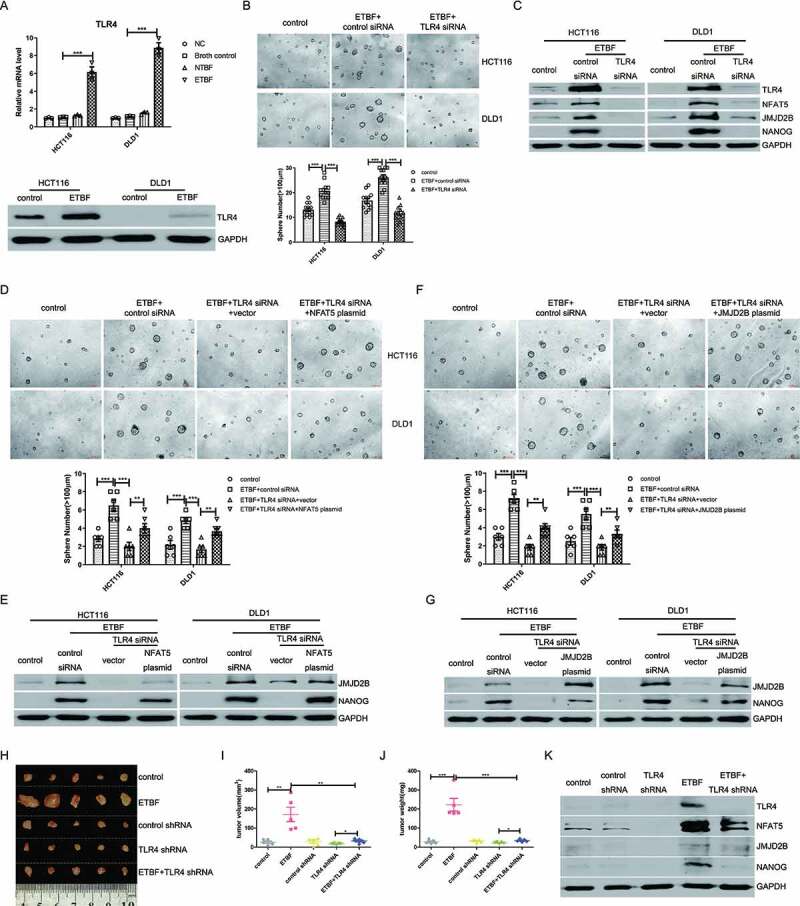
ETBF induces CRC cells stemness through Toll-like receptor 4 (TLR4). A, Real-time PCR and western blotting of TLR4 in HCT 116 and DLD1 cells co-cultured with ETBF (control: Broth control). B, ETBF-co-cultured HCT116 and DLD1 cells (500 cells/200 μL per well) with either scrambled control or TLR4 siRNA were subjected to tumorsphere formation assay, and the average number of tumorspheres (≥ 100 µm in diameter) was analyzed. C, Western blotting showing the levels of NFAT5, JMJD2B, and NANOG in HCT 116 and DLD1 cells co-cultured with ETBF and transfected with TLR4 siRNA. D, NFAT5 overexpression plasmid transfection could abolish the effect of TLR4 siRNA on the reduction of tumorsphere formation in HCT116 and DLD1 cells (500 cells/200 μL per well). E, Western blotting was performed to detect the levels of JMJD2B and NANOG in CRC cells. HCT116 and DLD1 cells were treated with TLR4 siRNA, co-cultured with ETBF, and transfected with the NFAT5 overexpression plasmid. F, Tumorsphere formation assays were performed. HCT116 and DLD1 cells (500 cells/200 μL per well) were treated with TLR4 siRNA, co-cultured with ETBF, and transfected with JMJD2B overexpression plasmid. G, Western blotting was performed to detect the expression of NANOG. HCT116 and DLD1 cells were treated with TLR4 siRNA, co-cultured with ETBF, and transfected with JMJD2B overexpression plasmid. H – J, Representative data of tumors (H) in nude mice bearing HCT116 cells (1 × 10^6^ cells) in different indicated groups. Statistical analysis of tumor volume (I) and tumor weight (J) in different groups was performed, n = 5/group. K, Western blotting showed the levels of TLR4, NFAT5, JMJD2B, and NANOG from the representative xenograft tumor tissues in different indicated groups. Data are expressed as the mean ± SEM from three independent experiments. Statistical significance was determined by ANOVA. **, *P < *.01; ***, *P < *.001.

The TLR signaling pathway is activated in response to *Bacteroides fragilis* (*B. fragilis*) intervention.^37,38^ Consistent with these findings, we found that TLR4 was highly expressed after ETBF infection (Figure 4A), whereas the mRNA levels of other *TLRs* that are located on the cell membrane were not notably affected (data not shown). To examine whether TLR4 participates in ETBF-induced stemness, we transfected CRC cells with TLR4 siRNA and co-cultured the cells with ETBF. We found that knockdown of TLR4 significantly reduced the formation of ETBF-induced tumorspheres and abolished the effect of ETBF on NFAT5, JMJD2B, and NANOG expression (Figure 4B, C). Moreover, ectopic expression of NFAT5 or JMJD2B in HCT116 and DLD1 cells significantly reversed the TLR4 siRNA-impaired stemness and dramatically attenuated the reduction of JMJD2B and NANOG expression caused by the TLR4 siRNA (Figure 4D-G). Furthermore, ETBF-induced tumorigenesis was suppressed by knocking down TLR4 in the CRC xenograft mouse model, as shown by reduced tumor weight and tumor volume (Figure 4H-J). In addition, knockdown of TLR4 downregulated NFAT5, JMJD2B, and NANOG expression in the xenograft tumor tissues (Figure 4K).

Taken together, these data suggested that the stemness-promoting effect of ETBF depends, at least in part, on TLR4 activation.

### The levels of ETBF, NFAT5, JMJD2B, and NANOG correlate with in patients with CRC

**Figure 5.**
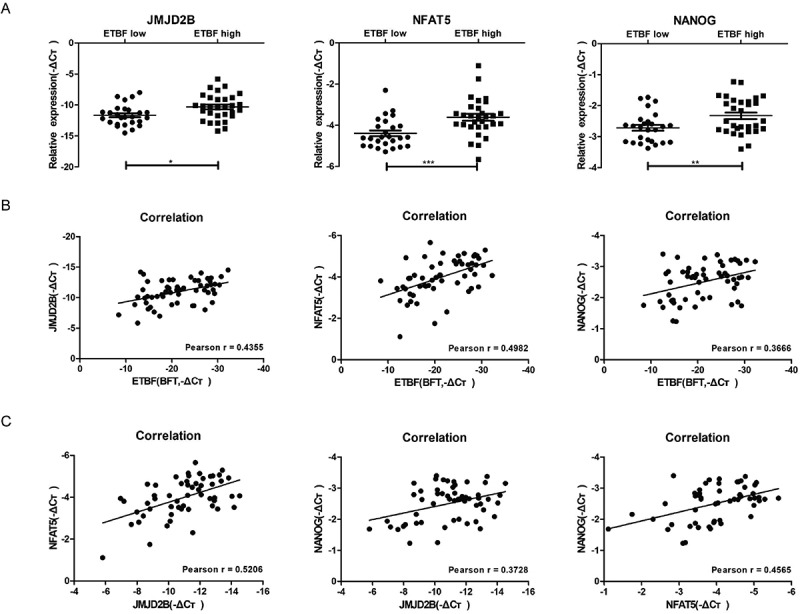
The correlation among levels of ETBF, *NFAT5, JMJD2B*, and *NANOG* in patients with CRC. A, Statistical analysis of *NFAT5, JMJD2B*, and *NANOG* mRNA expression in ETBF-low abundance and -high abundance groups in colorectal cancer tissues. B, Correlations of ETBF abundance, and *NFAT5, JMJD2B*, and *NANOG* levels in human colorectal cancer tissues. C, Correlation among *NFAT5, JMJD2B*, and *NANOG* levels in human colorectal cancer tissues. Data are expressed as the mean ± SEM. Statistical significance was determined by Mann-Whitney test (a) and Wilcoxon’s rank-sum test (b and c). *, *P < *.05; **, *P < *.01; ***, *P < *.001.

Given the interplay among ETBF, NFAT5, and JMJD2B, and their potential causal link to colorectal tumorigenesis in mouse models, we further tested these results in tissues from 56 CRC patients we studied above. The mRNA expression of *NFAT5, JMJD2B*, and *NANOG* significantly increased in the ETBF-high group (Figure 5A). Pearson rank sum test further showed that ETBF in CRC tissues correlated positively with *NFAT5, JMJD2B*, and *NANOG* (Figure 5B); *NFAT5* moderately correlated with *JMJD2B* and *NANOG* (Figure 5C). Thus, we demonstrated a link among ETBF, NFAT5, and JMJD2B and stemness in colorectal tumorigenesis.

## Discussion

**Figure 6.**
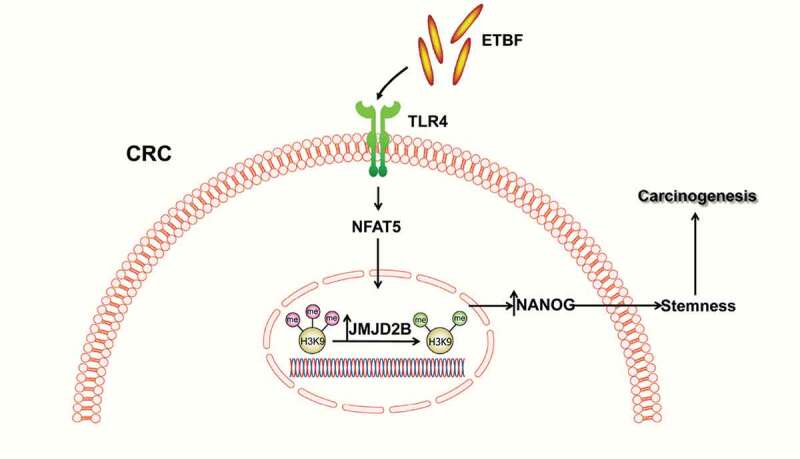
Schematic representation of ETBF-induced stemness in colorectal cancer via upregulating JMJD2B.

In the current study, we provided evidence that ETBF upregulates histone demethylase JMJD2B via a TLR4-NFAT5-dependent pathway, playing an important role in the stemness, which promotes colorectal carcinogenesis. Inhibition of JMJD2B expression *in vitro* and *in vivo* impairs ETBF-induced CSCs properties, through directly binding to and removing transcriptional repressive H3K9me3 marks on the *NANOG* promoter, which impedes CRC development (Figure 6). Moreover, the expression of *JMJD2B* is positively associated with ETBF abundance, *NFAT5* and *NANOG* expression in human CRC samples.

Microbiota alteration and cancer stem cells are important elements that contribute to CRC pathogenesis. Epidemiological studies have shown that the abundance of ETBF is significantly increased in patients with CRC.^3,39^ Animal model studies have also shown that ETBF can promote tumorigenesis by releasing *B. fragilis* toxin (BFT), a genotoxic compound. ETBF promotes colon tumorigenesis via upregulation of the polyamine catabolic enzyme, spermine oxidase (SMO), and reactive oxygen species (ROS). ETBF-induced colon tumorigenesis is Tregs/IL-2/Th17-dependent,^25^ and ETBF-triggered colon tumorigenesis is associated with an IL-17-driven myeloid signature characterized by subversion of steady-state myelopoiesis in favor of the generation of protumoral monocytic (MO)-myeloid-derived suppressor cells (MDSCs).^26^ In the present study, we demonstrated that ETBF promoted colorectal tumorigenesis in both the AOM sporadic CRC model and a nude mouse xenograft model. In line with these findings in mice, our human studies demonstrated that high levels of ETBF correlated positively with aggressive tumor-node-metastasis (TNM) stage. However, the mechanisms responsible for ETBF’s involvement in colorectal carcinogenesis are unclear. Our data showed that co-culture with ETBF could enhance the stemness of CRC cells using a sphere-forming assay. Stemness maintainers NANOG and SOX2 were greatly induced in ETBF-infected cells. Accordingly, treatment with ETBF increased the growth and the size of tumor organoids compared with those of the controls. Although several studies have demonstrated that the microbiota within the tumor microenvironment, such as acidophilic bacteria, *E. faecalis*, or nonpathogenic *E. coli*, could increase the stem cell activity of CRC, our data shows, for the first time, a potential causal link between ETBF and CRC development.

We subsequently explored the molecular mechanism by which ETBF induces CRC cells stemness. Changes in the intestinal microenvironment, such as dysbacteriosis, can cause histone modifications and chromatin structure alterations by recruiting or retrieving chromatin-modifying enzymes.^40,41^ H3K9me3 is a hallmark of gene transcriptional repression regions. Expression activation of the stemness maintainers NANOG and SOX2 are regulated by H3K9me3 demethylation.^42,43^ The histone demethylase JMJD2 is the only member of gene family that can remove H3K9me3, and its members include JMJD2A, JMJD2B, JMJD2C, and JMJD2D. Compelling evidence indicates that JMJD2B is overexpressed in human CRC tissue, and that it is implicated in various cellular processes, including apoptosis, cell cycle, invasion, DNA damage response, and metabolism to promote CRC progression.^44–47^ Furthermore, JMJD2B plays a critical role in self-renewal of ESCs and iPSC generation.^48,49^ Consequently, we hypothesized that ETBF promotes CRC stemness via JMJD2B. In support of this, we found that genetic inhibition of JMJD2B in ETBF-co-cultured cells inhibited sphere formation and NANOG expression. Furthermore, ETBF-induced pro-tumorigenicity effect was reversed using JMJD2B shRNA in the tumor bearing mouse models. ChIP data revealed high occupancies of H3K9me3 in the promoters of *NANOG*, and JMJD2B could transcriptionally upregulate the expression of *NANOG* by binding and removing the inhibitory H3K9me3 in the *NANOG* promoter region.

We also uncovered the mechanisms by which ETBF mediates JMJD2B upregulation. We used bioinformatics software to analyze the *JMJD2B* promoter sequence in detail and found that it contained multiple NFAT5 transcription factor binding sites, suggesting that NFAT5 is involved in the transcriptional regulation of *JMJD2B*. NFAT5 and NF-κB belong to the Rel family of proteins, which were first discovered in T cells. Subsequent studies have found that many tissues express NFAT5, such as the brain, kidney, and lung.^50^ In recent years, researchers have found that the function of NFAT5 is not limited to the renal medulla. This suggests that NFAT5 might also be involved in embryonic formation and development, liver detoxification, and tumor metastasis.^51–54^ Our functional studies revealed that NFAT5 targets JMJD2B, is selectively activated because of ETBF co-culture, and can biologically modulate CRC stemness *in vitro* and *in vivo*. Considering that the TLR signaling pathway is essential for *B. fragilis* infection,^55–57^ we demonstrated that the ETBF-induced genomic activation of NFAT5, JMJD2B, and NANOG depends on TLR4. Therefore, ETBF orchestrates TLR4, NFAT5, JMJD2B, and NANOG networks to exert biological control of CRC stemness. The potential virulence factors of *B. fragilis* which have been identified contain capsular polysaccharides (A-H),^58,59^ Lipopolysaccharide (LPS)^60,61^ and BFT.^5^ Recent studies have shown that the biological activities of *B. fragilis*-derived Polysaccharide A (PSA) and LPS are mediated by TLR4 activation.^60,62^ Similarly, Ahmadi et al. reported that *B. fragilis and B. fragilis*-derived outer membrane vesicles (OMVs, which contain bacterial components including LPS, outer membrane proteins, phospholipids, periplasmic components, DNA, RNA, hydrolytic enzymes and signaling molecules) both increase the mRNA levels of TLR4.^57^ BFT, key virulence factor of ETBF, binds to an uncharacterized cell surface receptor,^63,64^ triggering an array of signal transduction and contributing to key aspects of ETBF carcinogenic potential. However, until now, researchers have found that BFT may not directly induce Toll-like receptor activation.^65^ In our study, we stimulated HCT116 and DLD1 cells with ETBF and their mixed culture medium, which contain all potential virulence factors. Whether the ETBF-induced TLR4 activation is dependent on LPS, PSA, BFT or other identified/unidentified factors, awaits future studies.

In summary, our findings revealed that ETBF might act on CRC tumorigenesis via upregulating TLR4 and NFAT5, which subsequently transcriptionally upregulate JMJD2B, which increases NANOG expression by specifically demethylating promoter repressive H3K9me3, consequently promoting the CRC cells stemness. The current study provides a rationale for detecting and treating ETBF and identified JMJD2B as a promising anti-CRC target.

### Materials and methods

#### Patient specimens

Patients were diagnosed pathologically with CRC. The collection of CRC tissues was approved by the ethics committee of Renji Hospital, Shanghai Jiao Tong University School of Medicine (Shanghai, China). Informed consent was obtained from patients with CRC before sample collection in accordance with institutional guidelines. The relevant clinical and histopathological characteristics provided to the researchers were anonymized. All experiments were carried out in accordance with the provisions of the Helsinki Declaration of 1975.

#### Cell lines, bacterial strains, plasmids, and adenovirus

Human CRC cell lines HCT116 and DLD1 (ATCC, Manassas, VA, USA) were cultured in RPMI-1640 medium (Gibco, Grand Island, NY, USA) supplemented with 10% fetal bovine serum (FBS, Gibco). All cell lines were genotyped for identity by Beijing Microread Genetics Co., Ltd. Routine Mycoplasma testing was performed by MycoAlert Mycoplasma Detection Kit (Lonza, Basel, Switzerland, LT07-118) every 3 to 6 months. Cell lines were grown for no more than 10 passages in all experiments. Bacterial strains, nontoxigenic *Bacteroides fragilis* (NTBF, ATCC 25285), ETBF (ATCC 43860), *Fusobacterium nucleatum* (ATCC 25586), and *Clostridium symbiosum* (ATCC 14940) were cultured at 37 °C under anaerobic conditions (DG250, Don Whitley Scientific, West Yorkshire, UK) in brain heart infusion (BHI) broth supplemented with Yeast Extract, K_2_HPO_4_, Resazurin, L-cysteine, hemin, and Vitamin K1.^65^ Commensal *E. coli* strain DH5ɑ (CB101, Tiangen, Beijing, China) was cultured in Luria-Bertani medium for 12–16 h at 37 °C in shake cultivation at 220 rpm/min. For a single treatment, HCT116 and DLD1 cells were exposed to NTBF, ETBF, *F. nucleatum, C. symbiosum*, and *E.coli*, respectively, in penicillin/streptomycin-free RPMI-1640 (multiplicity of infection = 500) for 6 h. After 6 h, the medium containing bacterial strains was replaced with conventional cell culture medium. Plasmids pCMV-HA-NFAT5 and pCMV-HA-JMJD2B (wide-type and mutant) were purchased from Genechem (Shanghai, China). Short hairpin RNA (shRNA) adenovirus constructs targeting TLR4, NFAT5, and JMJD2B, and overexpression adenovirus constructs for JMJD2B were purchased from OBiO Technology (Shanghai, China).

#### Antibodies

Primary antibodies used were anti-TLR4 (Abcam, Cambridge, MA, USA, ab13556), anti-NFAT5 (Abcam, ab3446), anti-JMJD2B (Bethyl Laboratories, Montgomery, TX, USA, A301-478), anti-NANOG (Cell Signaling Technology, Danvers, MA, USA, 3580 S; Abcam, ab80892), anti-GAPDH (Kangcheng, Shanghai, China, 5G5), and anti-histone H3 (trimethyl K9) (Abcam, ab8898). Horseradish peroxidase (HRP)-conjugated goat anti-rabbit IgG (H + L) (Kangcheng; RB-035) was used as the secondary antibody.

#### Sphere forming assay*^66^*

Cells were trypsinized and plated onto Costar® 96-well Ultra-low attachment plates in serum-free medium consisting of serum-free Dulbecco’s modified Eagle’s medium (DMEM)/F12 (Gibco) with 1% B27 nutrient mixture (Thermo Fisher Scientific, Waltham, MA, USA) plus 20 ng/mL epidermal growth factor, 10 ng/mL fibroblast growth factor, 5 µg/mL insulin, and 0.4% bovine serum albumin. Formation of sphere-like structures was visible after 2 days, and images of each group were captured after 5 days. The number of spheres (diameter ≥ 100 μm) was calculated using Image J software (NIH, Bethesda, MD, USA).

#### RNA isolation, cDNA synthesis, and real-time PCR analysis

Total RNA was isolated using the TriZol Reagent (Life Technologies, Carlsbad, CA, USA) in accordance with the manufacturer’s instructions. For cDNA synthesis, 2 μg of quantified RNA was reverse transcribed using an SuperScript RT Reagent II Kit (Takara, Kusatsu, Shiga, Japan). Primers for qPCR were designed using PrimerBank or referred from the literature (Table 2). Real-time PCR was performed using a TB Green™ Premix Ex Taq™ II kit (Takara) on a StepOnePlus Real-Time PCR System (Applied Biosystems, Carlsbad, CA, USA). Gene expression was quantified by real-time PCR using *GAPDH* and 16 s RNA as housekeeping controls. The differential fold change in gene expression was calculated using the 2^(− ΔΔCt)^ method.^67^

**Table 2.** Primers uesd for PCR and real-time PCR.

Gene		Sequence	Product length (bp)
h *GAPDH*	Forward	GCATTGCCCTCAACGACCAC	78
	Reverse	CCACCACCCTGTTGCTGTAG	
h *CD133*	Forward	GGTCTGGCGAGCTAAGGGAA	217
	Reverse	GGGGAAGGCAAGCGTGTT	
h *CD44*	Forward	TTTGCATTGCAGTCAACAGTC	234
	Reverse	GTTACACCCCAATCTTCATGTCCAC	
h *LGR5*	Forward	TATGCCTTTGGAAACCTCTC	262
	Reverse	CACCATTCAGAGTCAGTGTT	
h *NANOG*	Forward	TCCAGCAGATGCAAGAACTCTCCA	131
	Reverse	CACACCATTGCTATTCTTCGGCCA	
h *OCT4*	Forward	TCAGCTTCCTCCACCCACTT	103
	Reverse	TATTCAGCCAAACGACCATCT	
h *SOX2*	Forward	ATGACCAGCTCGCAGACCTAC	107
	Reverse	TTGACCACCGAACCCATGGAG	
h *JMJD2B*	Forward	TCACCAGCCACATCTACCAG	68
	Reverse	GATGTCCCCACGCTTCAC	
h *NANOG* (for ChIP)	Forward	AGAAGTATTTGTTGCTGGGTTTGTCTTCAGG	199
	Reverse	GGCTCTATCACCTTAGACCCACC	
h *NFAT c1*	Forward	CTGCAGGACTCCAAGGTCAT	119
	Reverse	GGGATCTCAACCACCAGAGA	
h *NFAT c2*	Forward	ACCAGGAGTTCCAGCACATC	124
	Reverse	TGCTGAATGACTGTGGGGTA	
h *NFAT c3*	Forward	AGTTCCATCTTTGCCTGTGC	123
	Reverse	TATGTTTGTGGGATGGAGCA	
h *NFAT c4*	Forward	GGGCCCACTATGAGACAGAA	120
	Reverse	TGCCGATGAACATCTGTAGG	
h *NFAT 5*	Forward	CAAAGCCAACAAGGAACCAT	124
	Reverse	GTTGTTGTTGCTGCTGCTGT	
h *TLR4*	Forward	AGACCTGTCCCTGAACCCTAT	147
	Reverse	CGATGGACTTCTAAACCAGCCA	
m *Gapdh*	Forward	TGACCTCAACTACATGGTCTACA	85
	Reverse	CTTCCCATTCTCGGCCTTG	
m *Nanog*	Forward	CACAGTTTGCCTAGTTCTGAGG	86
	Reverse	GCAAGAATAGTTCTCGGGATGAA	
16 s	Forward	GGTGAATACGTTCCCGG	145
	Reverse	TACGGCTACCTTGTTACGACTT	
*bft*	Forward	GACGGTGTATGTGATTTGTCTGAGAGA	294
	Reverse	ATCCCTAAGATTTTATTATCCCAAGTA	
NTBF	Forward	TTCAACCTGATCGATCCGGAAGATCCG	1600
	Reverse	GCTGGTAGACTACCTGAGTAAGGAGTC	

#### Protein isolation and western blotting

Whole cell lysates were prepared by adding Radioimmunoprecipitation assay (RIPA) lysis buffer (Beyotime, Shanghai, China) with a protease inhibitor cocktail (Kangcheng). For xenograft tumor lysate preparation, snap frozen tumors were homogenized using a high-throughput tissue grinder (WonBio, Shanghai, China) before adding lysis buffer. Isolated total proteins were quantified using a bicinchoninic acid (BCA) Protein Assay Kit (Thermo Fisher Scientific), and equal amounts of lysates were resolved in appropriate SDS-PAGE gels, transferred onto 0.45-μm polyvinylidene fluoride (PVDF) membranes (Millipore, Bangalore, India), and then probed with primary antibodies. HRP-conjugated secondary antibodies and Supersignal West Pico chemiluminescent substrate (Thermo Fisher Scientific) were used for immunoreactive protein band detection using the ChemiDoc™ Imaging System (BIO-RAD, Hercules, CA, USA). GAPDH was used as the protein loading and transfer control.

#### RNA interference

Small interfering RNAs (siRNAs) specifically targeting TLR4, NFAT5, and JMJD2B were purchased from GenePharma (Shanghai, China) using the following sequences: JMJD2B: siRNA-1, 5′-GCGCAGAAUCUACCAACUU-3′ and siRNA-2, 5′-CAAAUACGUGGCCUACAUA-3′. These siRNAs were used as a pool for siRNA transfection. The other siRNAs were TLR4 siRNA: 5′-GCCGAAAGGUG

AUUGUUGUTT-3′; and NFAT5 siRNA: 5′-GCAACACAGTTTCAGACAA-3′. HCT116 and DLD1 cells were seeded at 30% confluence in six-well plates overnight before transfection and then transfected with 100 nM siRNA using the Lipofectamine™ 2000 Transfection Reagent (Thermo Fisher Scientific) in accordance with the manufacturer’ s instructions. A nonspecific siRNA was used as a negative control.

#### Chromatin immunoprecipitation (ChIP) assays

ChIP assays were conducted in accordance with the manufacturer’s protocol (Millipore). Briefly, cells were incubated with 1% formaldehyde for 10–15 min at 37°C. The cells were then lysed and sonicated. The samples were centrifuged at 13,000 rpm. The diluted supernatant was pre-cleared using 75 μL of protein A agarose beads. Then, the anti-JMJD2B and anti-H3K9me3 antibodies were immunoprecipitated with the crosslinked mixture overnight at 4°C. After a series of washes, the crosslinking was reversed and purified for PCR and real-time PCR analysis. The primer sequences for the human *NANOG* promoter for ChIP are shown in Table 2.

#### Dual-luciferase reporter assay

Sub-confluent HCT116 and DLD1 cells were seeded in 96-well plates and co-transfected with luciferase reporter plasmids expressing *NFAT5, JMJD2B* (wid-type and mutant), and Renilla luciferase (Generay, Shanghai, China). After incubation for 24 h, the cells were lysed and subjected to luciferase assays using the Dual-Luciferase Reporter Assay System (Promega, Madison, WI, USA) and FLUOstar® Omega multi-mode microplate reader (BMG Labtech, Offenburg, Germany).

#### AOM murine model

Conventional male C57BL/6 mice were bred in the Animal Laboratory at the Renji Hospital, Shanghai Jiao Tong University School of Medicine. In the mouse model of azoxymethane (AOM, Sigma-Aldrich, St Louis, MO, USA)-induced colorectal tumorigenicity, we gave antibiotics to the mice through drinking water, comprising 0.2 g/L ampicillin, neomycin, and metronidazole, and 0.1 g/L vancomycin (Sigma-Aldrich) daily for 2 weeks. After the last dose of antibiotics, the mice were injected with AOM intraperitoneally (10 mg/kg, once a week for 10 weeks) and gavaged with 1 × 10^9^ colony forming units (CFU) of NTBF or ETBF twice weekly for 20 weeks. The mice were killed 3 days after the last gavage.

#### Xenografts in nude mice

Five-week-old male BALB/c nude mice were housed under specific pathogen-free (SPF) conditions. To evaluate the role of ETBF in regulating stemness *in vivo*, 1 × 10^5^, 5 × 10^5^, and 1 × 10^6^ HCT116 cells were injected subcutaneously to establish separate CRC xenograft models. In the subsequent xenograft experiments, 1 × 10^6^ HCT116 cells were injected subcutaneously. One week after subcutaneous inoculation, the mice were randomly divided into different groups for different sets of experiments. The relevant viral vectors and ETBF (multiplicity of infection = 500) were given via multipoint intratumoral injection, twice a week for three weeks.

Tumor volumes were calculated as follows: (longest diameter) ×(shortest diameter)^2^ × 0.5. At the end point, the tumors were dissected and analyzed. All animal studies were conducted in accordance with the guidelines published in the Animal Ethics Committee.

#### Immunohistochemistry

Tumor tissues were fixed in 4% paraformaldehyde at room temperature for 24 h and then embedded in paraffin. Samples were cut into 4-μm sections, de-paraffined, and then rehydrated. Endogenous peroxidase activity was quenched with 3% H_2_O_2_ for 10 min, and the sections were washed and heated by a microwave in citrate buffer (Maxim, Fujian, China) for antigen retrieval. Then, sections were blocked with horse serum (Maxim) for 30 min and incubated with primary antibodies against NANOG at 4°C overnight and HRP-conjugated polyclonal anti-mouse/rabbit antibodies (Maxim) at room temperature for 30 min. Sections were developed with DAB buffer (Maxim) using standard protocols.

#### Generation and propagation of organoid cell cultures*^68^*

Isolated colorectal tumors from C57BL/6 mice were trypsinized for single-cell culture, mixed with Matrigel (Corning, NY, USA), and then placed in 24-well plates at 15000 cells per 50 μL of Matrigel per well. The Matrigel was allowed to polymerize at 37°C, and then basal culture medium (advanced DMEM/F12, Gibco) containing 1 U/mL penicillin (Gibco), 1 μg/mL streptomycin (Gibco), 10 mmol/L HEPES (Gibco), 2 mM Glutamax (Gibco), 1 × N2 supplement (Gibco), 1 × B27 supplement (Gibco), 50 ng/mL of murine EGF (Gibco), and 2.5 ng/mL amphotericin B (Sigma-Aldrich), 1 mmol/L N-acetylcysteine (Sigma-Aldrich). The culture medium was changed every 2 days, and organoids were passaged at 1:5 once a week.

#### Detection of the total bacteria, NTBF, and ETBF in mice stool samples

*Bacteroides fragilis* toxin gene (*bft*) was used to identify oncogenic ETBF. The primer sequences for NTBF, ETBF (*bft*) and 16 s are described in Table 2. Genomic DNA was extracted from mouse stool samples of equal weight using a QIAamp PowerFecal DNA Kit (QIAGEN, Hilden, Germany). DNA from each specimen was subjected to PCR to determine the amounts of total bacteria, NTBF, and ETBF. Each reaction contained 100 ng of DNA and was assayed in triplicate.

#### Detection of ETBF in human CRC tissue

Genomic DNA was extracted from human CRC tissue using a QIAamp Fast DNA Tissue Kit (QIAGEN) and subjected to real-time PCR to determine the amounts of ETBF. The primer sequences of ETBF (*bft*) and 16 s are described in Table 2. Each reaction contained 100 ng of DNA and was assayed in triplicate.

#### Statistical analysis

All statistical analysis were performed using GraphPad Prism6 software (GraphPad Software, Inc. La Jolla, CA, USA). Independent sample t test (unpaired Student’s t test and Mann-Whitney test) was conducted for the comparison of two conditions. Analysis of variance (ANOVA) was used for multiple comparisons. Wilcoxon’s rank-sum test was performed to evaluate the correlations of ETBF abundance, and *NFAT5, JMJD2B*, and *NANOG* levels. The χ^2^-test was used to evaluate the correlation between ETBF abundance and the clinicopathological factors of patients with CRCs. Values were considered significantly different at *P <* .05 and expressed as the mean ± SEM from three independent experiments.

## Supplementary Material

Supplemental Material
