# Supplementary material for: Enterotoxigenic Bacteroides fragilis induces the stemness in colorectal cancer via upregulating histone demethylase JMJD2B
Source: Gut Microbes. 2020 Jul 20;12(1):1788900. doi: 10.1080/19490976.2020.1788900 (PMC7524313; doi:10.1080/19490976.2020.1788900)
Supplement: Supplemental Material [file KGMI_A_1788900_SM0276.pdf]

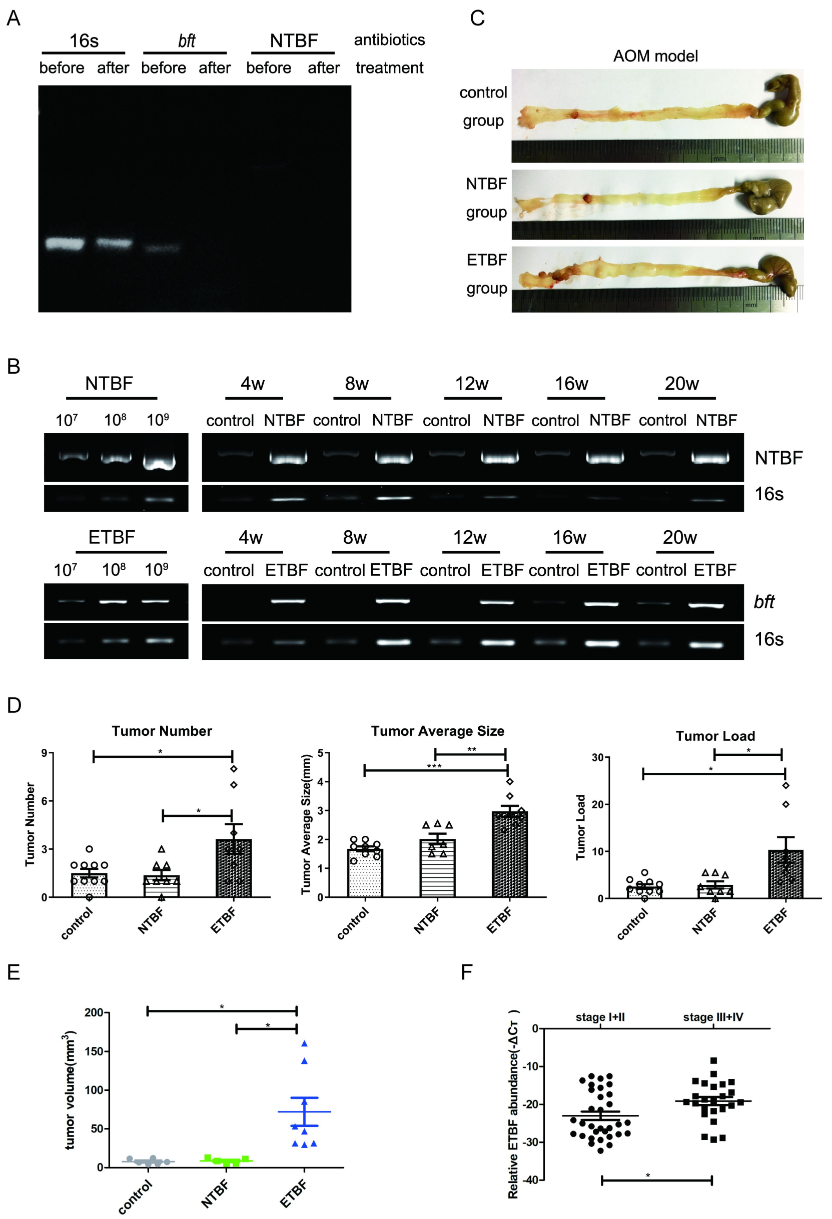

Supplementary Figure S1.

ETBF promotes colorectal tumorigenesis both in the AOM model and xenograft model. **A,** The level of NTBF and ETBF (*bft*) in stool samples of mice before and after receiving the antibiotics for 2-week, was determined by PCR analyses. The 16s RNA gene was used as an internal control. **B,** The levels of ETBF (*bft*) and NTBF in stool samples of mice during ETBF or NTBF feeding period, as assessed using PCR. The 16s RNA gene was used as an internal control. The levels of ETBF (*bft*) and NTBF in ETBF or NTBF liquid were used as positive controls. **C,** Representative intestinal morphologies of mice in the AOM model under different treatments; n =

8/group. **D,** Intestinal tumor numbers, tumor average size, and tumor load from eight mice treated with AOM only, eight mice treated with AOM + NTBF, and eight mice treated with AOM + ETBF. **E,** Tumor volume in the xenograft model treated with NTBF (n = 5) or ETBF (n = 5). **F,** ETBF abundance in different TNM stage groups of CRC patients. Data are expressed as the mean ± SEM from three independent experiments. Statistical significance was determined by ANOVA (D and E) and Mann-Whitney test (F). *, $P < 0.05$; **, $P < 0.01$; ***, $P < 0.001$.

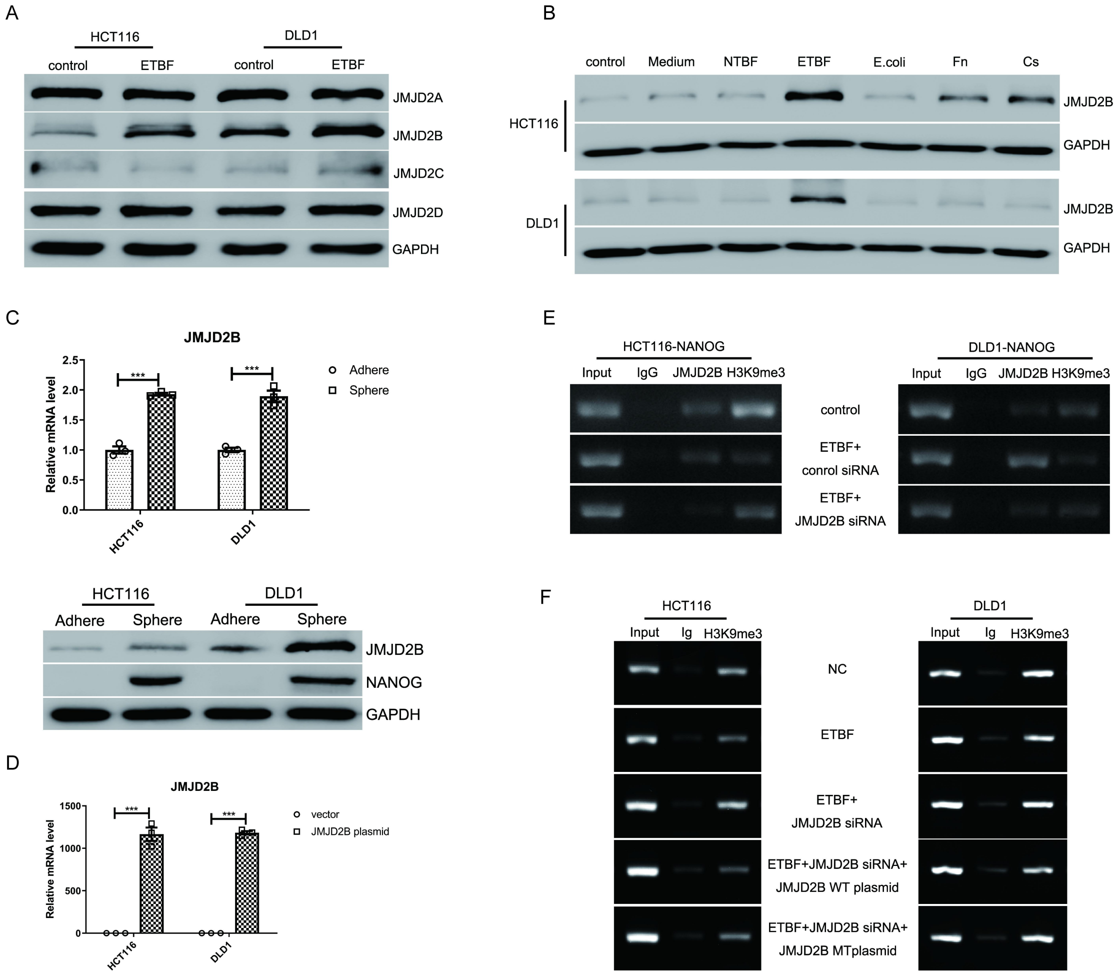

Supplementary Figure S2.

JMJD2B is involved in ETBF-mediated stemness. **A,** Western blotting was performed to detect the level of JMJD2 family members in CRC cells co-cultured with ETBF (control: Broth control). **B,** Western blotting was performed for JMJD2B levels in HCT116 and DLD1 cells co-cultured with ETBF, *E. coli*, *F. nucleatum*, and *C. symbiosum*. **C,** Real-time PCR and western blotting were performed in the tumorsphere cells and in the adherent cells to detect the mRNA and protein level of JMJD2B. **D,** JMJD2B expression was significantly increased in HCT116 and DLD1 cells. Real-time PCR was used to assess gene expression. **E,** Effect of JMJD2B on the occupancies of H3K9me3 in the promoters of *NANOG* in HCT116 and DLD-1 cells by a ChIP assays (PCR). **F**, Analysis of the levels of H3K9 tri-methylation binding to the *NANOG* promoter in HCT116 and DLD-1 cells to test whether the decrease in H3K9me3 intensity was depended on the lysine de-methylation activity of JMJD2B

directly (PCR). Data are expressed as the mean ± SEM from three independent experiments. Statistical significance was determined by unpaired Student′s t test. **, $P < 0.01$; ***, $P < 0.001$.

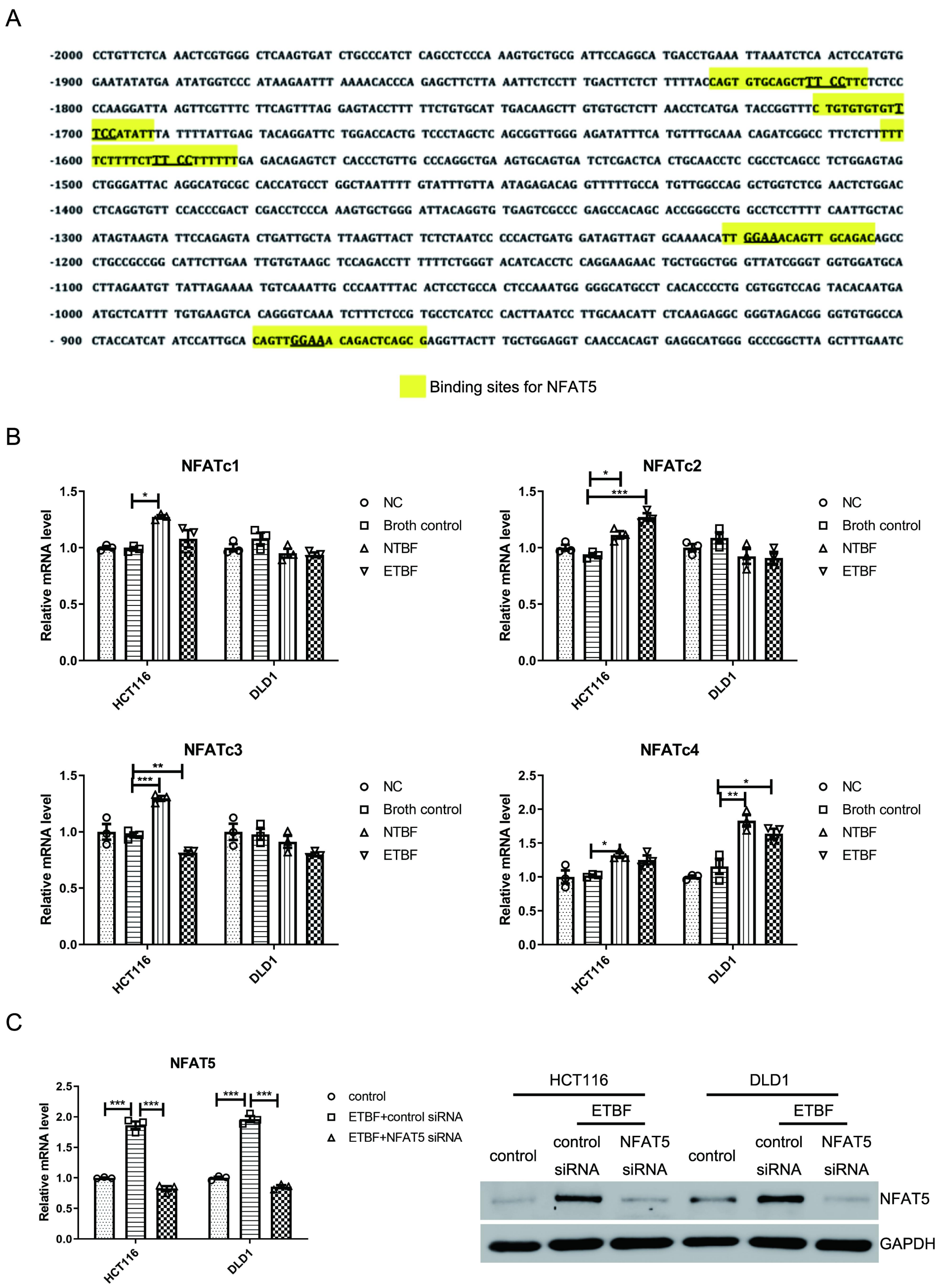

Supplementary Figure S3.

ETBF promotes the expression of JMJD2B through NFAT5, related to Figure 3. **A,** The promoter region of *JMJD2B* containing multiple NFAT5 transcription factor binding sites (highlighted in yellow). **B,** The expression of *NFAT c1-c4* were measured using real-time PCR in CRC cells co-cultured with ETBF. **C,** NFAT5 expression was induced in HCT116 and DLD1 cells co-cultured with ETBF and suppressed after transfection with NFAT5 siRNA. Real-time PCR and western blotting were used to assess gene and protein expression, respectively. Data are expressed as the mean ±

SEM from three independent experiments. Statistical significance was determined by ANOVA . *, $P < 0.05$; **, $P < 0.01$; ***, $P < 0.001$.
